# Antiseizure monotherapy with imepitoin or phenobarbital in feline idiopathic epilepsy: a multicenter, single-blinded, randomized and placebo-controlled study

**DOI:** 10.3389/fvets.2026.1770972

**Published:** 2026-02-06

**Authors:** Marios Charalambous, Andrea Tipold, Holger A. Volk, Anna Knebel, Enrice-Ina Hünerfauth, Thilo Von Klopmann, Stefan Rupp, Bart J. G. Broeckx, Sofie F. M. Bhatti

**Affiliations:** 1 Clinic for Small Animals, University of Veterinary Medicine Hannover, Hannover, Germany; 2 Clinic for Small Animals Hofheim, Hofheim, Germany; 3 Laboratory of Animal Genetics, Faculty of Veterinary Medicine, Ghent University, Merelbeke-Melle, Belgium; 4 Small Animal Department, Faculty of Veterinary Medicine, Ghent University, Merelbeke-Melle, Belgium

**Keywords:** cats, drugs, efficacy, management, safety, seizures

## Abstract

In feline idiopathic epilepsy (IE), the options for antiseizure medications (ASMs) remain limited with no licensed drugs available in cats in Europe. This study aimed to evaluate and compare efficacy and safety of imepitoin and phenobarbital through a multicenter, single-blinded, randomized, placebo-controlled trial. A total of 37 cats were included in this study. The study treatment evaluation period lasted for 15 weeks. In the imepitoin group (*n* = 16), monthly seizure frequency was significantly reduced (*p* = 0.028; mean pre-treatment, 6.1; mean post-treatment, 3.0), though monthly seizure days (*p* = 0.055; mean pre-treatment, 4.4; mean post-treatment, 2.6) and number of cluster seizures (*p* = 1.00; mean pre-treatment, 0.9; mean post-treatment, 0.3) did not show significant changes. The responder rate (i.e., > 50% reduction in seizure frequency post treatment) was 62%. In the phenobarbital group (*n* = 10), treatment led to a significant reduction in monthly seizure frequency (*p* = 0.0026; mean pre-treatment, 8.1; mean post-treatment, 1.3) and seizure days (*p* = 0.0011; mean pre-treatment, 5.7; mean post-treatment, 0.5), but not in the number of cluster seizures (*p* = 0.82; mean pre-treatment, 1.3; mean post-treatment, 0.4). The responder rate was 90%. When compared, the reduction in seizure days was significantly higher for phenobarbital compared to imepitoin (*p* = 0.036), while no significant difference was found for seizure frequency (*p* = 0.13) and responder rate (*p* = 0.1). The time to first seizure event after starting treatment was significantly longer in the phenobarbital group compared to imepitoin (*p* = 0.047) and placebo (*p* = 0.0017), but not between imepitoin and placebo (*p* = 0.078). Adverse effects of mild to moderate severity were observed in 90% of the phenobarbital group (primarily sedation and ataxia) and 88% of the imepitoin group (primarily ataxia and increased ALT activity). Both phenobarbital and imepitoin demonstrated efficacy and safety in feline IE. While seizure frequency reduction did not differ significantly between treatments, phenobarbital was associated with a prolonged time to first seizure event after treatment initiation. Adverse effects were common but the majority of these effects were mild to moderate and transient.

## Introduction

Research on feline idiopathic epilepsy (IE) management is considerably less extensive than that of canine epilepsy, yet epileptic seizures represent a prevalent neurological condition among cats, with an estimated occurrence rate of 0.5–3.5% within clinical populations (1–3). Studies indicate that between 21 and 59% of cats presenting with recurrent seizures may be diagnosed with IE (4–6).

The approach to managing IE in dogs and cats significantly differs, primarily due to variations in the safety profiles of antiseizure medications (ASMs) across the two species (7, 8). While it is generally recognized that the potential benefits of ASMs in terms of their effectiveness can outweigh their adverse effects, some of the ASMs used in dogs can pose safety concerns in cats. Consequently, both the efficacy and the safety profile of the drug should be carefully assessed when selecting the most suitable ASMs for managing feline patients (9).

In veterinary medicine, established guidelines exist for the management of epilepsy (10, 11) and seizure emergencies in dogs (12). In contrast, for feline patients, we only have official guidelines for the treatment of seizure emergencies, even though strong recommendation and level of evidence still remain limited (12). Regarding treatment guidelines for feline epilepsy, comprehensive and detailed recommendations remain underdeveloped. The range of ASMs available for use in cats is limited compared to dogs, primarily due to a lack of robust evidence, including high-quality studies and clinical expertise, as well as safety concerns, particularly with medications such as oral diazepam (e.g., hepatic necrosis) and potassium bromide (e.g., respiratory disease) (13). Imepitoin and phenobarbital are very commonly used as first choice ASMs in veterinary patients, however, they have different levels of evidence and regulatory approval in cats with epilepsy.

Imepitoin is a low-affinity partial agonist at the benzodiazepine site of the Gamma-Aminobutyric Acid Type A receptor (GABA(A)) receptor, enhancing inhibitory neurotransmission and increasing seizure threshold, with currently limited feline data suggesting generally good tolerability and mainly mild, transient adverse effects such as ataxia, anorexia or gastrointestinal signs. In contrast, phenobarbital is a long-acting barbiturate that potentiates GABA(A) receptor–mediated inhibition and reduces neuronal excitability, and it is widely regarded as a first-line treatment for feline epilepsy in clinical practice (13, 14). Phenobarbital use is contraindicated or requires caution in cats with hepatic disease or significant systemic illness, and its common dose-dependent adverse effects include sedation, ataxia, and polyphagia, which often diminish over time; rare but serious adverse effects such as hepatotoxicity or hematologic abnormalities might occur and necessitate serum concentration and laboratory monitoring (13, 14).

Even though imepitoin is a well-established ASM licensed in Europe for the management of IE in dogs (in the US is officially approved for the treatment of noise aversion in dogs), in cats it is often used as an off-label ASM. Data on its use in feline patients is limited to non-existing, particularly regarding its efficacy. To date, only a single pilot study has assessed the tolerability of imepitoin in both healthy cats and those with IE, reporting favorable tolerability outcomes (14). Overall, there is a lack of large-scale, well-designed studies in veterinary medicine evaluating the efficacy and safety profile of imepitoin and phenobarbital in cats with IE.

To the best of our knowledge, this study represents the first blinded, randomized, placebo-controlled trial evaluating and comparing the efficacy and safety of two ASMs, imepitoin and phenobarbital, for the treatment of feline IE. The primary objective of this study was to evaluate whether imepitoin could serve as a viable alternative to phenobarbital for the management of feline IE, with the overarching goal of improving quality of life for affected cats. Specifically, we aimed to assess both the efficacy and safety profile of imepitoin, and to compare these outcomes to those observed with phenobarbital and placebo. Given the limited therapeutic options currently available for feline IE, identifying additional safe and effective treatments is of significant clinical importance. This study was designed to provide evidence to support the potential incorporation of imepitoin into the treatment repertoire for feline patients, thereby offering veterinarians and owners an alternative strategy for chronic seizure management in cats.

## Materials and methods

### Population

Inclusion criteria:

1. Drug-naïve cats: Cats with a history of ≥2 isolated epileptic seizures, cluster seizures (CS; ≥2 seizures within 24 h), or ≥1 status epilepticus (defined as seizure activity lasting >5 min) within 8 weeks prior to inclusion.
2. Normal interictal findings: Cats were required to have a normal interictal physical and neurological examination as well as unremarkable results from hematology and blood biochemistry (including serum Alanine Transaminase (ALT), Alkaline Phosphatase activity (ALP), Aspartate Aminotransferase (AST), ammonia, bile acids, glucose, electrolytes, blood urea nitrogen (BUN), and creatinine). While serology for toxoplasmosis and FeLV/FIV and urinalysis were desirable, they were not mandatory as they were considered of lower clinical priority for the current study.
3. Age-specific requirements:

- For cats aged 0.5–7 years at the onset of epileptic seizures, a normal brain magnetic resonance imaging (MRI) and cerebrospinal fluid (CSF) analysis were desirable to rule out structural intracranial lesions, but not mandatory.
- For cats younger than 0.5 years and older than 7 years, brain MRI and CSF analysis were mandatory for inclusion in addition to the already described requirements, especially since, based on clinical experience as well as current evidence, cats beyond these age margins at seizure onset might be more likely to have structural epilepsy (15).

Exclusion criteria:

1. Evidence of hippocampal pathology on MRI, or clinical features suggestive of temporal lobe epilepsy (e.g., focal seizures with orofacial automatisms such as salivation, facial twitching, lip smacking, chewing, licking, and swallowing), if no MRI was available.
2. The presence or development of a concomitant disease during the study that could result in neurological signs or interfere with study outcomes (e.g., renal or liver failure, diabetes mellitus).
3. Pregnant or lactating female cats.
4. Life-threatening conditions likely to preclude study completion (e.g., congestive heart failure).
5. Prior treatment with imepitoin or phenobarbital for more than five consecutive days immediately before inclusion.

A signed consent form was obtained from the owners before the cats’ inclusion in the study. The study was approved by the Universities Ethical Committees (Belgium, EC 2017/123; Germany, LAVES TV 19/3262).

### Study design

A multicenter, single-blinded, randomized controlled clinical trial was designed to evaluate the efficacy and safety of imepitoin monotherapy compared to phenobarbital monotherapy and placebo in drug-naïve cats with IE. Cat owners remained blinded, while the treating veterinary surgeon knew about the medication after randomization. Drug-naïve cats were randomized^1^ using sealed randomization envelops into one of three groups and treated for 15 weeks with either 30 mg/kg imepitoin PO twice daily (Group A), 2.5 mg/kg phenobarbital PO twice daily (Group B), or placebo PO twice daily (Group C). All the medications were made to look identical. Cats assigned to the placebo group were monitored for seizure occurrence. If one seizure occurred post-placebo treatment initiation, the placebo trial was terminated, and the cat was randomly reassigned to Group A or Group B. The evaluation period was restarted for these cats to assess their response to the new treatment modality. Group assignments were performed using sealed randomization envelopes and the owners remained blinded to the medication.

#### Evaluation period

**Figure 1:**
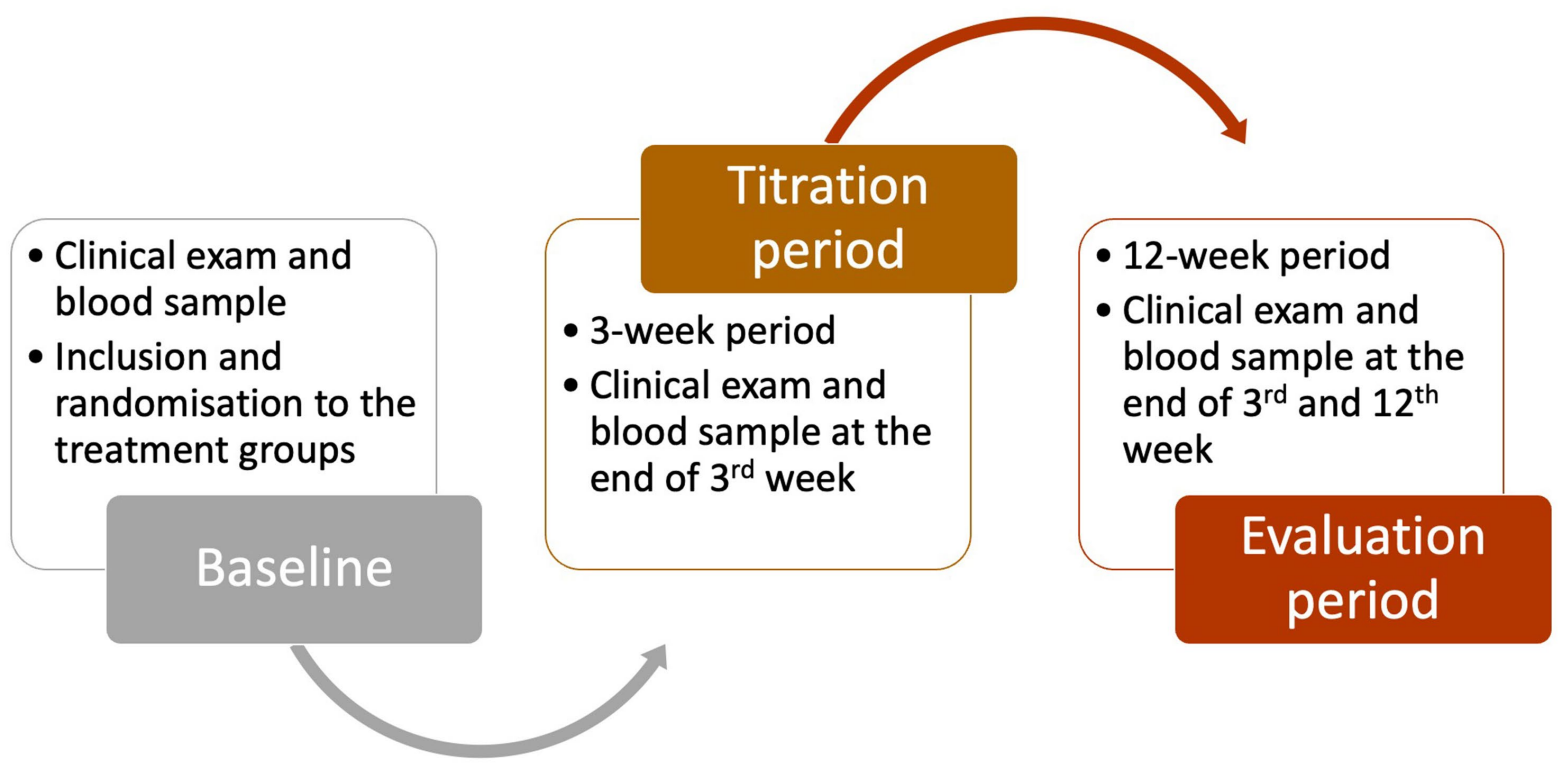
Flow chart of the study process and time points.

An evaluation period of 12 weeks was required for each cat (Figure 1). To maintain a blinded protocol, all medications were dispensed in unlabeled High-Density Polyethylene (HDPE) bottles to prevent owners from identifying the medication. For phenobarbital-treated cats, serum phenobarbital levels were also measured when taking blood samples, and dose adjustments were made as necessary to achieve target serum concentrations (23–30 mg/L). Blood samples were taken from all cats in all groups. The owners remained blinded because they were informed in advance that blood sample would be taken for laboratory tests and that the medication dose might change following blood examination in any of the treated groups, while the dose change does not indicate any specific medication. Therefore, the 12-week evaluation period commenced after the initial 3-week titration phase, resulting in a total treatment duration of 15 weeks in all groups. Overall, after inclusion, owners kept seizure logs and presented their cats for clinical, neurological, and blood evaluations at 3 weeks (during titration phase) and again at six and 15 weeks after the titration period (during evaluation phase).

Blood samples were collected for hematology and biochemistry analyses, including serum glucose, electrolytes, and liver enzymes and kidney function at all time points (3, 6 and 15 weeks). In the phenobarbital group, phenobarbital serum levels were measured to ensure optimal dosing. Doses of ASM remained unchanged during the evaluation unless severe or life-threatening adverse effects (e.g., marked sedation, ataxia, or disorientation lasting over 7 days) occurred.

#### Study endpoints

The study endpoints included:

1. A 100% increase in seizure frequency.
2. Development of severe or life-threatening adverse effects (e.g., profound sedation, ataxia, polyuria/polydipsia/polyphagia, or idiosyncratic reactions such as leukopenia, thrombocytopenia, anemia, lymphadenopathy, skin eruptions, or coagulopathy).

### Outcome assessment

#### Efficacy

Prior to inclusion, investigators and owners documented the number of epileptic seizures occurring over at least the previous 12 weeks as a baseline. Each cluster seizure (CS) event and individual seizures within a cluster were recorded.

The primary outcomes included:

1. Monthly seizure frequency (MSF).
2. Monthly seizure day frequency (MSDF).
3. CS events.

Cats with 50–100% reduction in MSF were defined as responders. Cats with 0 to <50% reduction or an increase in MSF were defined as non-responders.

#### Safety profile

Adverse effects were recorded in the study files at the re-check appointments with the owners. In addition, the onset, duration, and severity of the adverse effects also were reported. Regarding the severity, a score was was assigned independently for each reported adverse effect ranging from 1 (very mild) to 5 (very severe) with the aim to quantify the degree of severity.

### Statistical analysis

All statistical analyses were conducted in R version 4.3.2. A Wilcoxon signed rank test was conducted to compare MSD, MSDF, and number of CS events pre-treatment (before any medication started) versus post-treatment (after the end of the 15-week treatment period) within each of the two treatment groups (imepitoin and phenobarbital). If an effect was found, a subsequent analysis was conducted to assess whether the treatment effect differed significantly between the two treatment groups by testing the significance of the interaction of treatment and period (pre/post) in a linear mixed model with animals as random effects, using a likelihood ratio test. A Fisher exact test was used to compare the number of individuals observed with a seizure during the follow-up period for the treatments and placebo groups (phenobarbital, imepitoin, and placebo were compared). A Kruskal–Wallis test was used to compare the time to the first seizure after initiation of treatment in these three groups, followed by a post-hoc Wilcoxon rank sum test when significant. For those individuals where the data was censored (i.e., that did not have a seizure by the end of the study period), this was approximated by setting the interval to study period plus 1 day (i.e., 106 days). Throughout all analyses, significance was set at *α* ≤ 0.05. A Bonferroni-correction for multiple testing was applied, whenever necessary.

## Results

### Population

Details about the population characteristics are provided in Table 1.

**Table 1:** Study population characteristics.

Number (*n*) of cats included	Total number (*n* = 37) Imepitoin (*n* = 16; 43%) Phenobarbital (*n* = 10; 27%) Placebo (*n* = 11; 30%)
Number (*n*) of cats with advanced investigation	Brain MRI and cerebrospinal fluid analysis (*n* = 26; 70%)
Seizure type	Generalised tonic–clonic without focal seizures (*n* = 27; 73%) Generalised tonic–clonic with focal seizures (*n* = 10; 27%)
Breed	European shorthair (*n* = 26; 69%) British shorthair (*n* = 4; 11%) Domestic shorthair (*n* = 4; 11%) Oriental shorthair (*n* = 1; 3%) Ragdoll (*n* = 1; 3%) Norwegian (*n* = 1; 3%)
Age	Median, 3.8 years (mean, 4.5; range, 1–5)
Sex	Female neutered (*n* = 22; 60%) Male neutered (*n* = 15; 40%)

### Efficacy

**Figure 2:**
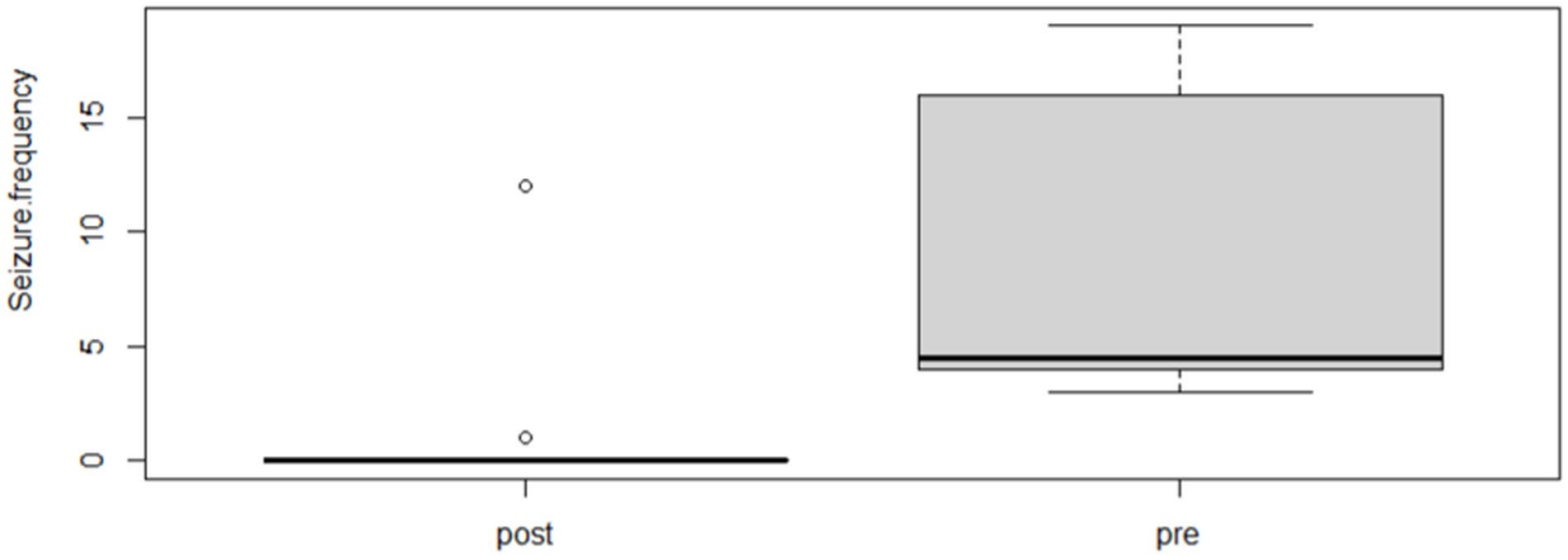
Phenobarbital MSF boxplot showing the difference between pre- and post-treatment (*p* = 0.0026; mean pre, 8.1; mean post, 1.3).

**Figure 3:**
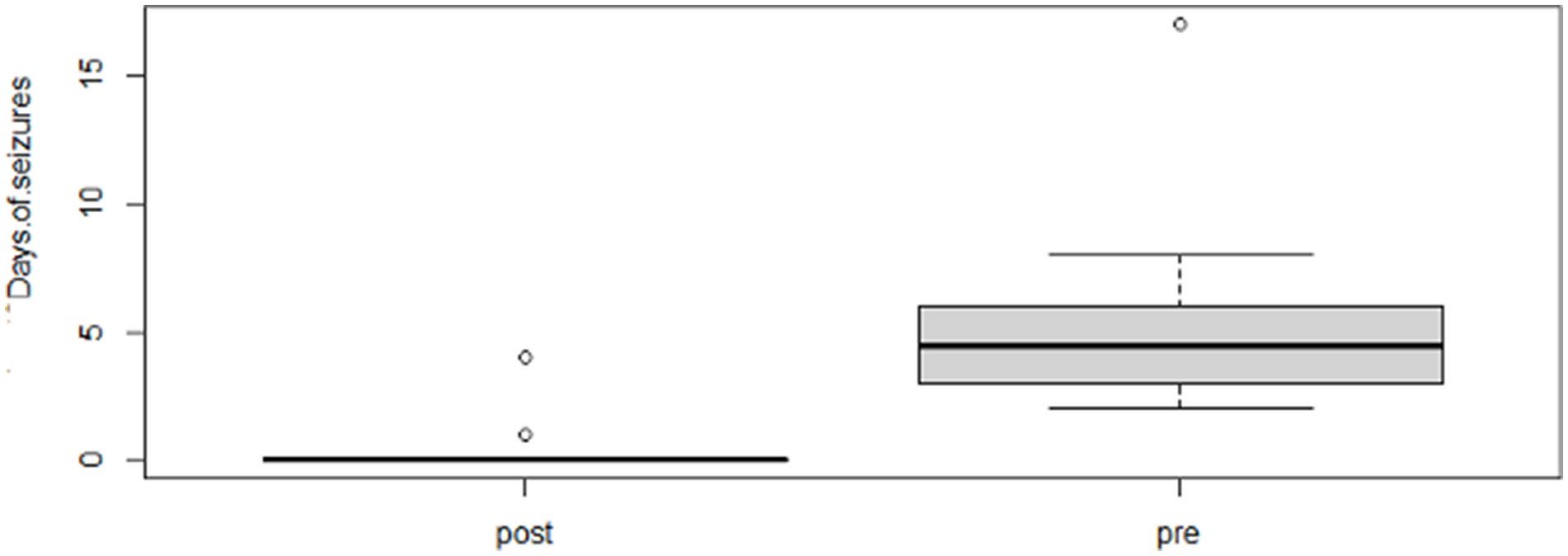
Phenobarbital MSD boxplot showing the difference between pre- and post-treatment (*p* = 0.0011; mean pre, 5.7; mean post, 0.5).

**Figure 4:**
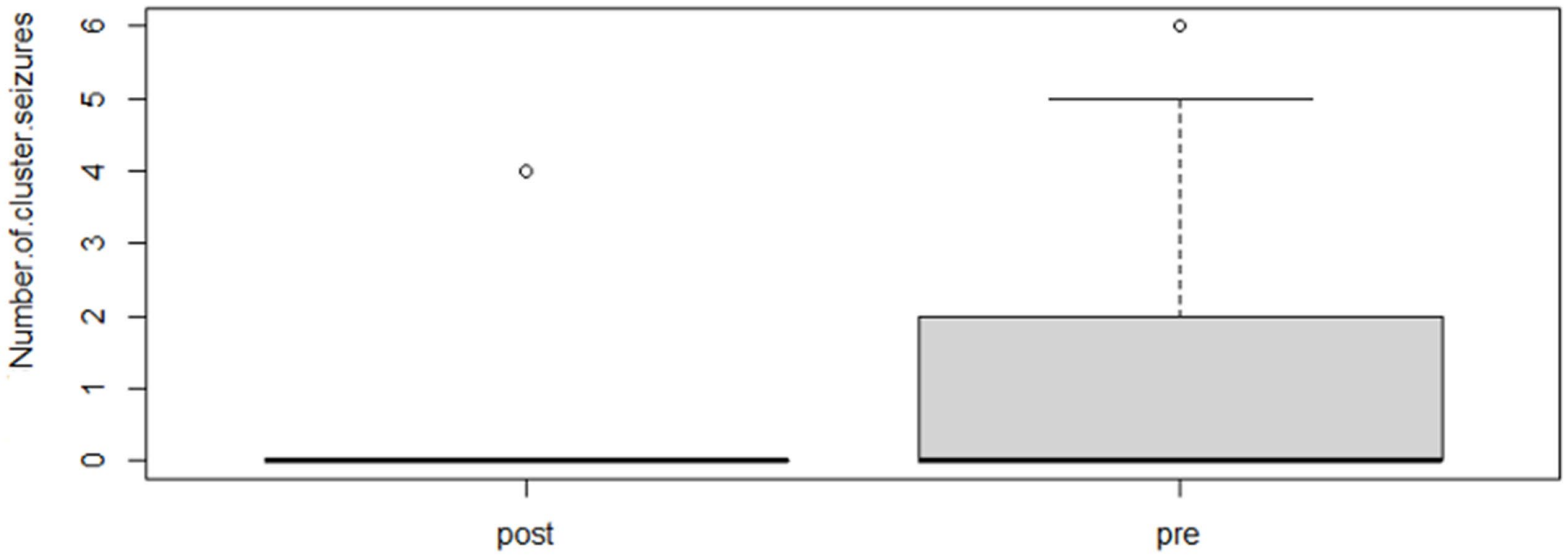
Phenobarbital number of CS events boxplot showing the difference between pre- and post-treatment (*p* = 0.82; mean pre, 1.3; mean post, 0.4).

Following the completion of the study, within the phenobarbital group, there was a significant reduction of MSF (*p* = 0.0026; mean pre, 8.1; mean post, 1.3; Figure 2) and MSDF (*p* = 0.0011; mean pre, 5.7; mean post, 0.5; Figure 3), but not for number of CS (*p* = 0.82; mean pre, 1.3; mean post, 0.4; Figure 4) compared to baseline. Overall, 90% (*n* = 9) were considered as responders. Seizure freedom occurred in 80% (*n* = 8). The non-responders (10%, *n* = 1) showed a < 50% reduction in MSF. The median phenobarbital serum levels were 28 mg/L (mean, 27 mg/L; range, 14.5–41.9 mg/L). The median phenobarbital dose was 2.2 mg/kg BID (mean, 2.15 mg/kg; range, 1.3–3 mg/kg). In two cats (responders), the dosage had to be increased or decreased due to serum levels below or above the reference range, respectively.

**Figure 5:**
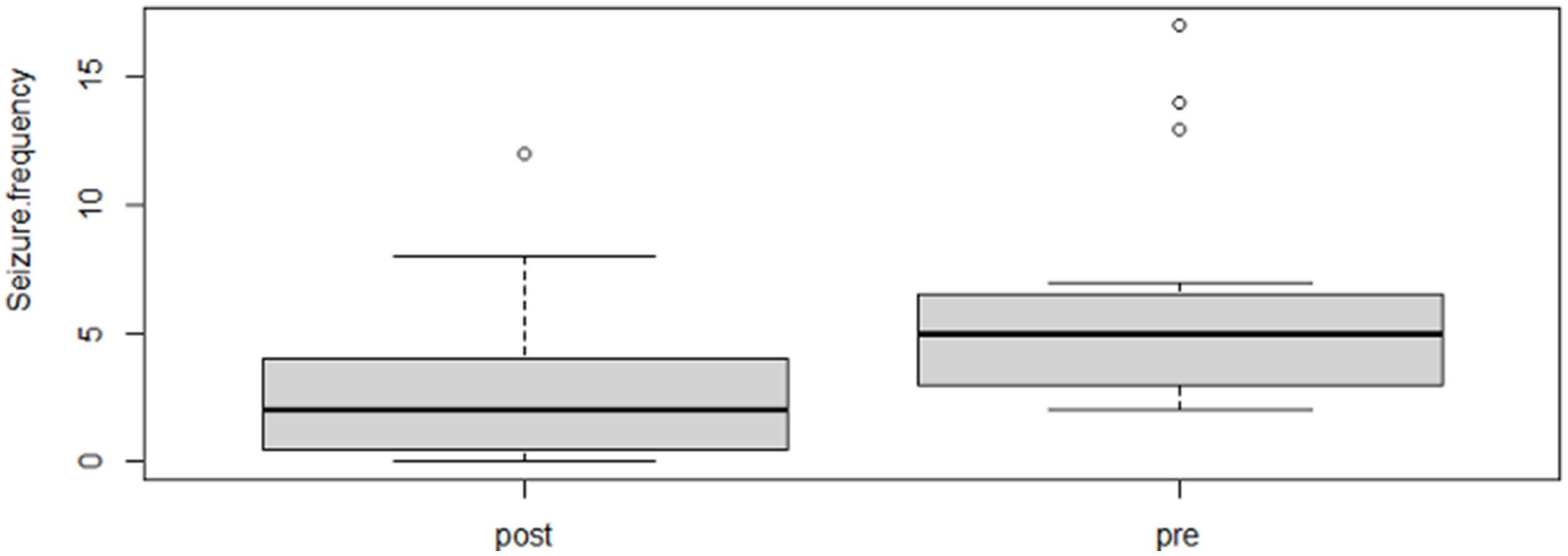
Imepitoin MSF boxplot showing the difference between pre- and post-treatment (*p* = 0.028; mean pre, 6.1; mean post, 3.0).

**Figure 6:**
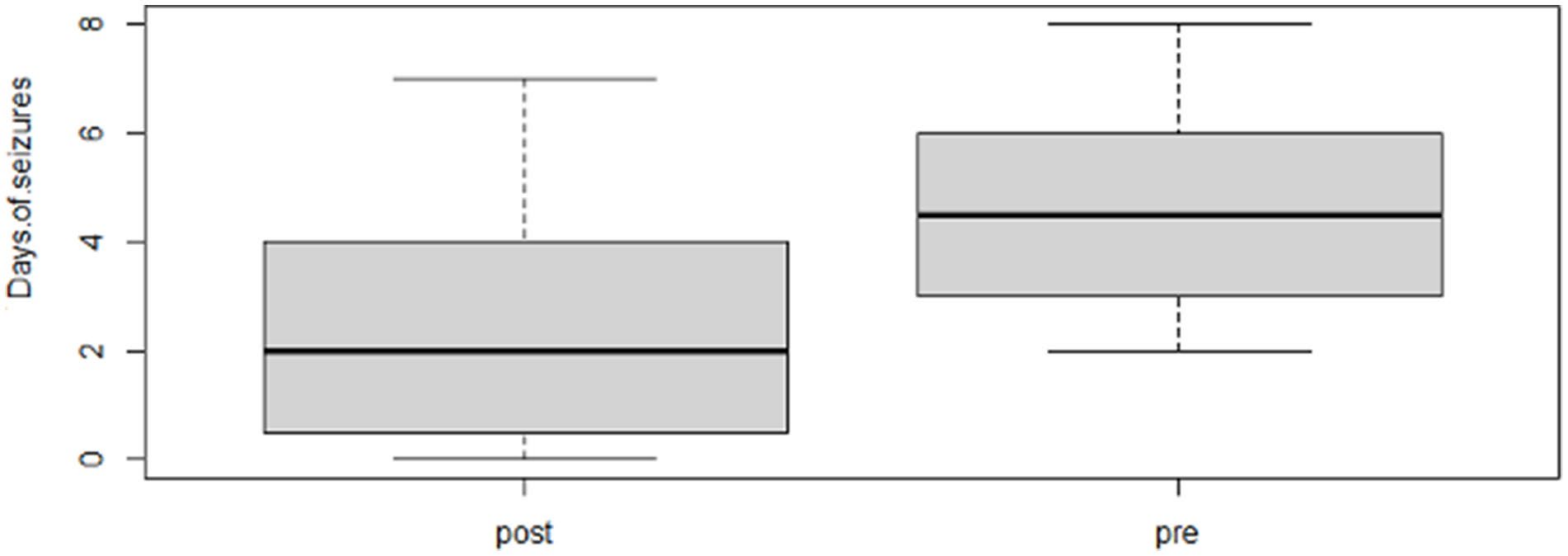
Imepitoin MSD boxplot showing the difference between pre- and post-treatment (*p* = 0.055; mean pre, 4.4; mean post, 2.6).

**Figure 7:**
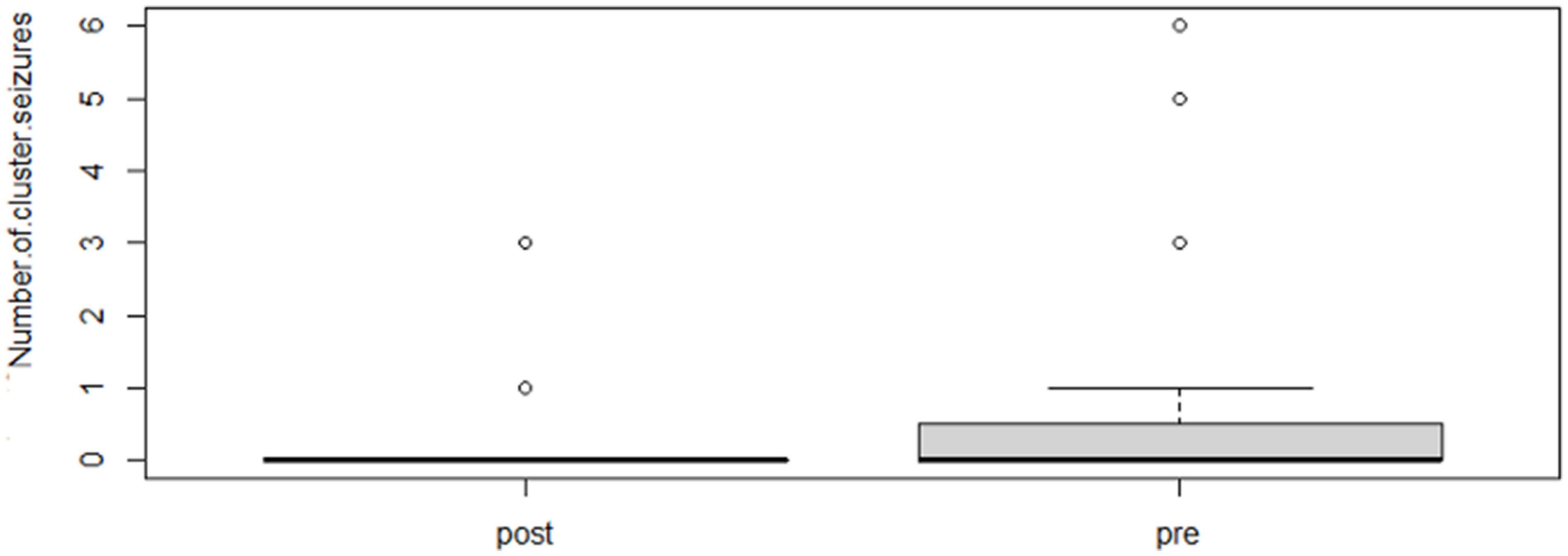
Imepitoin number of CS events boxplot showing the difference between pre- and post-treatment (*p* = 1.00; mean pre, 0.9; mean post, 0.3).

Within the imepitoin group, there was a significant reduction of MSF (*p* = 0.028; mean pre, 6.1; mean post, 3.0; Figure 5), but not for MSDF (*p* = 0.055; mean pre, 4.4; mean post, 2.6; Figure 6) and number of CS (*p* = 1.00; mean pre, 0.9; mean post, 0.3; Figure 7) compared to baseline. Overall, 62.5% (n = 10) were considered as responders. Seizure freedom occurred in 25% (n = 4). The non-responders (37.5%, *n* = 6) showed either a < 50% reduction in MSF (19%; *n* = 3) or an increase in MSF occurred (19%, *n* = 3) cats. The median imepitoin dose was 30 mg/kg BID (mean, 28; range, 18–31). In three cats, the dosage had to be decreased, following owner request, due to severe adverse effects, i.e., lethargy and ataxia (*n* = 1) or lethargy, ataxia, weight loss, reduced appetite and vomiting (*n* = 1), or sedation, ataxia and increased liver enzymes (ALT, AST, ALP, bilirubin; *n* = 1).

The difference in effect between phenobarbital and imepitoin was significant for MSDF, where the reduction was more pronounced in the phenobarbital group (*p* = 0.036). No significant difference was found for MSF (*p* = 0.13) and response rate (*p* = 0.1).

Regarding the time to first epileptic seizure event after ASM start, there was a significant difference in the number of cats that had a seizure observed during the follow-up period (*p* < 0.001) across all groups. The time to first seizure was significantly different between phenobarbital and imepitoin (favoring phenobarbital; *p* = 0.047), phenobarbital and placebo (favoring phenobarbital; *p* = 0.0017), but not between imepitoin and placebo (*p* = 0.078).

### Safety profile

**Figure 8:**
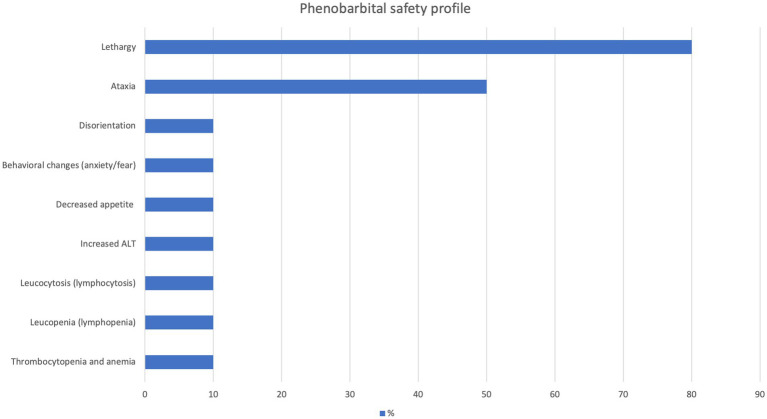
Adverse effects of phenobarbital.

In the phenobarbital group, the prevalence of adverse effects was 90% (*n* = 9). The most common adverse effects were lethargy and ataxia (see Figure 8 for details). The median severity score of phenobarbital adverse effects was 4, 2, and 2 at the first, second, and third control appointments, respectively. The overall median severity score of adverse effects was 2.5 (mean, 2.8; range, 1–5). By the end of the study, adverse effects completely subsided in 60% (*n* = 6) of the cats treated with phenobarbital.

**Figure 9:**
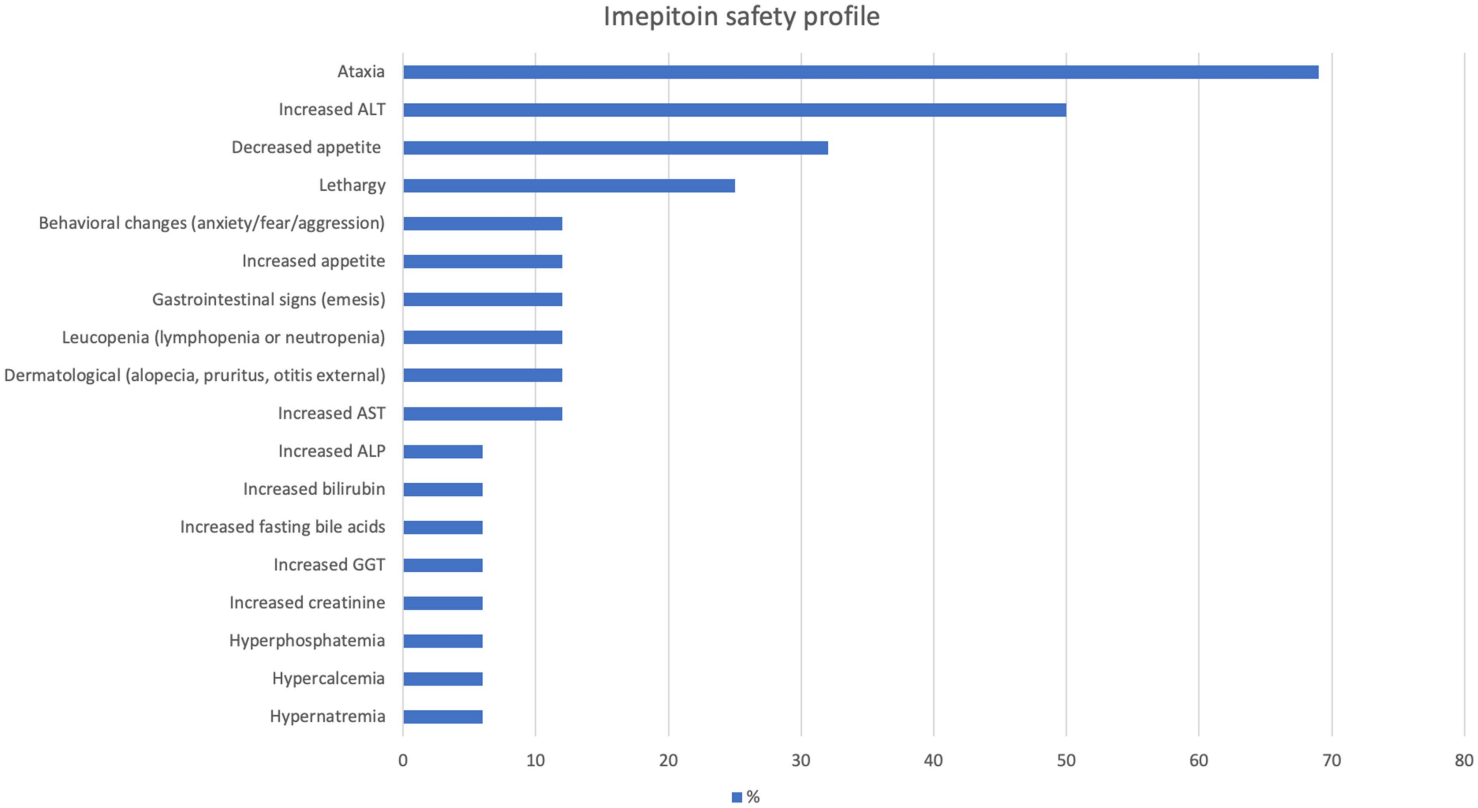
Adverse effects of imepitoin.

In the imepitoin group, the prevalence of adverse effects was 88% (*n* = 14). The most common were ataxia and increased ALT (see Figure 9 for details). The median severity score of imepitoin adverse effects was 2, 2, and 2 at the first, second and third control appointments, respectively. The overall median severity score of adverse effects was 2 (mean, 2.2; range, 1–5). In 25% (*n* = 4) of the cats, the dose had to be reduced from 30 mg/kg BID to a range of 20–25 mg/kg BID because of severe adverse effects including ataxia and lethargy; the severity of adverse effects was reduced after the dosage reduction and was tolerable by the cats and owners. In these four cats that dosage was decreased, only one was considered a responder regarding seizure frequency reduction. By the end of the study, adverse effects completely subsided in 38% (*n* = 6) of the cats treated with imepitoin.

In the placebo group, no adverse effects were reported.

## Discussion

To the authors’ knowledge, this is the first blinded randomized placebo-controlled study evaluating and comparing the effects of imepitoin and phenobarbital in cats with IE.

Our results show that both imepitoin and phenobarbital can be reasonable options and effective in feline IE. Within each group, both of the medications showed significant reductions in MSF pre-treatment compared to post-treatment. When the antiseizure effect between imepitoin and phenobarbital was compared, no significant difference in MSF reduction and response rate was found. However, phenobarbital showed a significantly longer time to first seizure post-treatment when compared to imepitoin. Regarding the safety profile, the majority of the cats showed adverse effects in both groups with mild to moderate severity which subside over time.

Pharmacological treatment remains the primary approach for managing epilepsy in cats. While substantial advancements have been made in diagnosing, classifying, characterizing, and especially treating epilepsy in dogs, progress in feline epilepsy remains relatively limited (13). Managing epileptic seizures often requires long-term, continuous care and ongoing research to enhance therapeutic outcomes and improve the quality of life for affected animals (13, 16). A systematic review conducted by the author(s) in 2018 evaluating the efficacy and safety profile of ASMs in cats revealed that the level of evidence supporting treatment options for feline epilepsy, particularly the efficacy of ASMs was generally weak or absent (13). Similarly, the evidence behind the management of seizure emergencies in cats, including CS and status epilepticus, was also scarce (12). However, our current clinical trial adds to evidence regarding the efficacy of imepitoin and phenobarbital as monotherapies in cats with IE. Our study confirmed that both imepitoin and phenobarbital are effective and safe treatment options, expanding the options of well-established therapies available for cats with epilepsy.

Regarding safety profiles, the adverse effects of ASMs can generally be classified into predictable and dose-dependent, idiosyncratic, unpredictable, and dose-independent (9, 13, 17). Based on the systematic review in cats (13), imepitoin was identified as the safest ASM, followed by levetiracetam and phenobarbital, then zonisamide and pregabalin; potassium bromide and diazepam were considered the least safe. While most safety assessments were supported by weak evidence, imepitoin’s safety profile was substantiated by a stronger level of evidence. Based on this, the majority of the reported cases did not exhibit adverse effects with phenobarbital or imepitoin monotherapy (13). Conversely, our study revealed that most cats experience adverse effects of mild to moderate severity with either monotherapy.

An interesting observation was the impact of monotherapy on liver function. In the imepitoin monotherapy group, elevated ALT was the most frequently observed adverse effect (50% of the cases), with increased ALP, GGT, bilirubin, gamma-glutamyl transferase, and fasting bile acids also detected (6% of the cases). Although no alternative causes were identified during the study that could account for these elevated enzyme values, it remains unclear whether imepitoin was directly responsible for these changes. These findings suggest a potential hepatic effect of imepitoin in cats. This contrasts with a previous study that examined the safety profile of imepitoin in cats and did not find any impact of the medication on the liver (14). However, that study only included a small number of healthy non-epileptic cats. Consequently, periodic assessment of blood biochemistry, with or without complete blood count and electrolyte analysis, is recommended for cats undergoing imepitoin monotherapy. Conversely, phenobarbital did not significantly increase hepatic enzyme activity in cats, except for a single case of elevated ALT, aligning with previous research findings (13, 18).

Blood dyscrasias were observed with both monotherapies, though phenobarbital was associated with more severe and diverse blood dyscrasias, including severe leukopenia, thrombocytopenia, and anemia (prevalence, 10%), necessitating treatment discontinuation. In contrast, imepitoin was linked to only mild leukopenia (prevalence, 12%) which did not necessitate discontinuation. Therefore, a complete blood count, ideally in conjunction with a biochemistry profile and serum phenobarbital concentration measurement, is recommended approximately 3 weeks after initiating phenobarbital therapy (i.e., time needed for phenobarbital to reach steady serum levels in cats). Further blood monitoring may not be necessary unless systemic signs develop, adverse effects persist or worsen, or dose adjustments are made.

Establishing a correlation between ASM dosage or serum concentration and the incidence of adverse effects in feline patients has historically proven challenging (13). This difficulty may be attributed to the hypothesis that adverse effects in cats do not follow a dose- or serum concentration-dependent pattern, or alternatively, that available data have been insufficient to identify a potential association between these variables (13). In a previous study, only 33% of cats with a phenobarbital serum concentration exceeding 30 μg/mL exhibited elevated ALT or ALP levels beyond the reference interval, with no other abnormalities detected in bloodwork (18). Although the objective of our study was not to specifically investigate the impact of serum drug concentrations or dosages on the occurrence and severity of adverse effects, no definitive relationship could be identified in our feline population.

This study was designed incorporating blinded assessments, randomization, and placebo and ASM control groups. However, several limitations must be acknowledged. First, the relatively small number of cats may limit the ability to draw robust conclusions. The authors encountered difficulties in recruiting cats with IE, which may be attributed to a potentially lower population of cats with IE (in contrast to dogs) as well as lower inclination among feline owners in some countries to consult specialists, often relying on primary veterinarians who may treat these cases without a specialist referral. Additionally, the moderate follow-up period may restrict our ability to assess the long-term efficacy and safety of the treatments. Furthermore, some cats proved challenging to treat, which could be due to misclassification or issues with patient and owner compliance. Misclassification and poor compliance were identified as causes of pseudoresistance in 12 and 9% of cases, respectively, in dogs diagnosed with IE (19). In our feline study, although owners reported adherence to the treatment schedule, difficulties in administering oral medications to some cats were reported, potentially leading to missed doses. Regarding misclassification, not all cats underwent brain MRI scans or CSF analysis to rule out intracranial pathology, including temporal lobe epilepsy and limbic encephalitis. Even for those that were tested, subtle pathologies may have been overlooked, particularly in centers utilizing low-field MRI or when inflammatory changes were mild, as reported previously (20, 21). In addition, it has also been reported in some studies that a vast majority of cats with hippocampal necrosis exhibit normal brain MRI scans and CSF (20, 22), potentially leading to false negatives and an underestimation of ASM efficacy and safety. Structural epilepsy, including temporal lobe epilepsy and limbic encephalitis, is more difficult to treat in cats compared to IE, particularly in the absence of additional targeted therapies, which could impact ASM response (20, 21). This is especially relevant for imepitoin, which has been approved for use in dogs with IE but lacks sufficient evidence or recommendations for its use in cats with structural epilepsy. Conversely, phenobarbital has been assessed and suggested as a potent ASM for both feline and canine structural epilepsy, highlighting the need for more targeted studies in cats. Lastly, the authors used a scoring approach with the aim to provide a semi-quantitative measure of adverse-effect severity and to facilitate standardized reporting across cases. Although the system was inherently subjective and dependent on clinician judgment, it was implemented to enhance comparability and interpretability of clinical observations. The authors acknowledge that this method is not without limitations, including potential inter-observer variability; however, it provides a pragmatic framework for contextualizing the relative severity of adverse effects as assessed by the attending clinician.

In conclusion, both imepitoin and phenobarbital demonstrated efficacy and safety in feline IE. While seizure frequency reduction did not differ significantly between treatments, phenobarbital was associated with a higher responder rate and prolonged time to first seizure event during the treatment period. Adverse effects were common but the majority was moderate and transient.

## Data Availability

The raw data supporting the conclusions of this article will be made available by the authors, without undue reservation.

## Footnotes

1
www.random.org
